# Phylogenomics supports a single origin of terrestriality in isopods

**DOI:** 10.1098/rspb.2024.1042

**Published:** 2024-10-30

**Authors:** Jessica A. Thomas Thorpe

**Affiliations:** ^1^ Wellcome Sanger Institute, Wellcome Genome Campus, Hinxton, Cambridge CB10 1SA, UK

**Keywords:** phylogenetics, Isopoda, phylogenomics

## Abstract

Terrestriality, the adaptation to life on land, is one of the key evolutionary transitions, occurring numerous times across the tree of life. Within Arthropoda, there have been several independent transitions: in hexapods, myriapods, arachnids and isopods. Isopoda is a morphologically diverse order within Crustacea, with species adapted to almost every environment on Earth. The order is divided into 11 suborders with the most speciose, Oniscidea, including terrestrial isopods such as woodlice and sea-slaters. Recent molecular phylogenetic studies have challenged traditional isopod morphological taxonomy, suggesting that several well-accepted suborders, including Oniscidea, may be non-monophyletic. This implies that terrestriality may have evolved multiple times. Current molecular hypotheses, however, are based on limited sequence data. Here, I collate available genome and transcriptome datasets for 36 isopods and four peracarid crustaceans from public sources, generate assemblies and use 970 single-copy orthologues to estimate isopod relationships and divergence times with molecular dating. The resulting phylogenetic analyses support monophyly of terrestrial isopods and suggest conflicting relationships based on nuclear ribosomal RNA sequences may be caused by long-branch attraction. Dating analyses suggest a Permo-Carboniferous origin of isopod terrestriality, much more recently than other terrestrial arthropods.

## 1. Introduction

Isopoda is a large and morphologically diverse order within the arthropod class Crustacea, comprising over 10 000 species across 132 families and 11 suborders (electronic supplementary material, figure S1) [1]. The most speciose suborder is Oniscidea, the group containing terrestrial woodlice and sea-slaters (>3700 species [2]). Adapted to almost every environment on Earth, isopods occupy a wide range of habitats, from tropical and temperate waters to polar regions. Marine isopods subsist between the sublittoral zone and the deep sea [3] associated with ecosystems such as kelp forests [4], coral reefs [5], rocky shores, sandy sediments and even hydrothermal vents [6]. Freshwater isopods can be found in both open and subterranean water sources [7–9]. Amphibious littoral isopods occupy shores and coastal regions, and while terrestrial isopods predominantly inhabit humid environments, some are secondarily aquatic in both fresh- and saltwater and others have adapted to life at high altitudes or even arid deserts [10,11]. Isopods also vary substantially in size and feeding behaviour. The smallest isopods, at below 0.2 mm, are meiofaunal [12], whereas giant, deep-sea isopods can reach 50 cm long [13]. Terrestrial isopods are detritivores and browsers, feeding on decaying wood and vegetation. Many aquatic isopods fill a similar niche, consuming dead and decaying plant and animal matter in the water. Some of these scavengers may also feed on slow-moving sea creatures [14]. Other marine isopods are filter-feeders, sieving detrital and planktonic food particles from the water, while some are predators of smaller aquatic life. Several groups are parasitic, with different suborders adapted to different host taxa, such as fish or Crustacea [15–17].

Isopods are one of several groups of arthropods (alongside hexapods, arachnids and myriapods) to have successfully colonized land. While the evolutionary route to terrestriality in arthropods is debated (via freshwater or littoral intermediates), the taxonomy of Oniscidea has long been considered a model of how terrestrial taxa could evolve from marine ancestors. Woodlice possess many adaptations essential to non-aquatic life, for example, a water-resistant cuticle, water-transport system, respiratory pleopods, carrying live young in a brood-pouch, and behavioural adaptations such as aggregation and conglobation [18]. Oniscidea has traditionally been divided into five sections, with the more derived terrestrial groups (Crinocheta, Synocheta and Microcheta) possessing more complex adaptations that facilitate life on land. For example, within the Crinocheta, several families possess pleopodal lungs (two, three or five pairs depending on the family) [19,20]. In contrast, the ‘earliest’ terrestrial isopods (Tylida, Diplocheta) are predominantly littoral and display behavioural and morphological traits associated with aquatic life [21]. For example, *Ligia*, a littoral sea-slater within Diplocheta, has a simple water-transport system, brood-pouch and open respiratory pleopods, and has been hypothesized to represent the amphibious, marine ancestor of woodlice [22]. Synocheta and Microcheta are considered intermediate, inhabiting highly humid, often subterranean, habitats [21,23].

The first phylogenetic analyses of Isopoda based on molecular data suggested that Oniscidea might not be monophyletic ([24–26]; electronic supplementary material, figure S2), and instead, littoral oniscids might be more closely related to marine suborders within Isopoda, adapting convergently to life on land. For example, Lins *et al*. [25] collated publicly available isopod sequence data (small (18S) and large (28S) nuclear ribosomal RNA (rRNA) subunits and mitochondrial cytochrome c oxidase I (COI)) and recovered Oniscidea as paraphyletic. The two oniscid clades Diplocheta (comprising littoral genus *Ligia* and freshwater-associated *Ligidium*) and Tylida (littoral *Tylos* and forest-dwelling *Helleria*) grouped with Phreatoicidea (Gondwana-distributed freshwater isopods), separate from the remaining Oniscidea (Crinocheta+Synocheta), albeit with extremely low phylogenetic support. Dimitriou *et al*. [26] investigated relationships across the five oniscid superfamilies with 18S, 28S and two nuclear protein-coding genes. Their analysis, demonstrating robust phylogenetic support, recovered *Ligia* alone outside Oniscidea, in a clade with other marine isopods, while remaining members of Diplocheta and Tylida were placed in a monophyletic Oniscidea. However, sampling of non-terrestrial isopods was limited, so the closest marine relatives of *Ligia* and relationships between other isopod suborders remain unresolved.

Across the other isopod suborders, molecular data have also indicated paraphyly of several accepted groups. Phylogenies based on nuclear rRNA genes have suggested non-monophyletic Asellota (the second-most speciose suborder including deep-sea isopods and freshwater pond-lice) [27], Cymothooidea (parasites of fish) [28], Sphaeromatidea (sea-pills and sand-skaters) [29] and Anthuroidea (elongate, predatory ‘wormpods’) [25], in addition to Oniscidea. The position of the root among the earliest branching nodes within Isopoda is also unclear. Molecular dating analyses recover one of the three parasitic groups, Cymothooidea, Epicaridea (crustacean parasites) or Gnathiidea (juvenile ‘pranzia’ are facultative fish parasites, sexually dimorphic adults likely non-feeding) near the root of the tree [25,28], whereas data from fossils and morphology suggest Asellota or Phreatoicidea are the oldest isopods, placed outside ‘Scutocoxifera’; the clade comprising the remaining suborders [1,30,31]. Analyses of whole mitochondrial genomes (mitogenomes) have also been applied to isopod phylogenetics (e.g. [32,33]) recovering Asellota or Phreatoicidea as the earliest branching groups. However, factors such as nucleotide composition, GC-skew and outgroup choice seem to impact which suborder is closest to the root and affect the monophyly of several other groups (Oniscidea, Epicaridea and Cymothooidea) [32,33]. Morphological studies are predominantly in agreement regarding the monophyly of most suborders (exceptions being Oniscidea [34] and Cymothooidea [1,31]). However, higher level relationships have been subject to multiple taxonomic revisions (see [1] for a summary of historical and recent studies); the most recent and perhaps well known being the replacement of Flabellifera (comprising Cymothooidea, Sphaeromatoidea (sea-pills), Seroloidea (sand-skaters) and Limnoriidea (tiny, marine wood-boring ‘gribbles’)) with Cymothoida (comprising Cymothooidea, Anthuroidea, Gnathiidea and Epicaridea, demoted to superfamily level) [1]. Furthermore, several elusive morphologically disparate, taxon-poor suborders have not yet been placed confidently with either molecular or morphological data (subterranean freshwater Tainisopidea [7,8] and Calabozoidea [9]), while Phoratopidea comprises only two specimens [1].

Genome and transcriptome data are now being deployed to resolve uncertain evolutionary relationships across Arthropoda (e.g. [35,36]). While a comprehensive dataset for Isopoda does not yet exist, many isopod genomic and transcriptomic datasets are publicly available. This study uses 970 single-copy orthologues across 36 isopod genomic and transcriptomic datasets to infer a phylogeny for Isopoda. In contrast to previous molecular analyses, this phylogeny shows monophyly of Oniscidea. Other relationships recovered include the separation of the parasitic isopod clades Epicaridea and Cymothooidea. A separate analysis of traditional phylogenetic marker genes indicates previous results may have been misled by long-branch attraction biases in nuclear rRNA sequences. Molecular dating suggests that terrestrial isopods made the transition to land during the Permian or Carboniferous, much more recently than other terrestrial arthropods.

## 2. Methods

### (a) Phylogenomic dataset

Genome and transcriptome datasets were collated for 36 isopods, one amphipod, one cumacean and two tanaid outgroups. This dataset included 21 terrestrial oniscideans (13 Crinocheta, five Synocheta, two Tylida and one Diplocheta), and 15 marine and freshwater isopods (four Asellota, one Epicaridea, four Cymothooidea, two Valvifera, three Sphaeromatidea and one Limnoriidea) (electronic supplementary material, table S1). Where data were available for more than one species in a given genus, the species with highest gene completeness was selected (see below). FASTQ files were downloaded from the European Nucleotide Archive (https://www.ebi.ac.uk/ena/browser). This phylogenomic dataset includes 6 of 11 isopod suborders. The remaining five: Phreatoicidea, Microcerberidea, Calabozoidea, Tainisopidea and Phoratopidea currently lack genomic data. Also missing are superfamilies Gnathiidea, Anthuroidea and Seroloidea, and within terrestrial isopods, *Mesoniscus* (Microcheta) and *Ligidium* (Diplocheta).

Raw reads were trimmed to remove low-quality bases (‘-q 20’) with *fastp* v. 0.23.2 [37]. For genomic datasets, trimmed reads were assembled into contigs with *SPAdes* v. 3.15.5 (kmers, k = 21,33,55,77,99,127) [38]. A scaffolded assembly for *Tylos granuliferus* was obtained from the authors [39]. Transcriptome datasets were assembled into contigs with *Trinity* v. 2.8.5 [40]. The longest gene isoforms were extracted with a custom Python script (https://github.com/jessthomasthorpe/Isopod_Phylogenomics_MS). For each taxon, single-copy orthologues from *arthropoda_obd10* (comprising 1013 genes) were retrieved from assembled contigs with *BUSCO* v. 5.4.2 (Benchmarking Universal Single Copy Orthologs [41]) (electronic supplementary material, table S1). Amino acid and nucleotide FASTA files were generated with *busco2fasta.py* (https://github.com/lstevens17/busco2fasta), with a cut-off of 50% taxonomic coverage, yielding a total of 970 BUSCO sequences. To assess the effect of alignment methods, amino acid BUSCOs were aligned with both *MAFFT* v. 7.520 and *FSA* v. 1.15.9 [42,43]. Alignments were inspected to verify that detected isoforms were the same across taxa, else the least common isoform was removed. To examine the effects of trimming, each set of alignments were either trimmed with *trimAl* v. 1.4.rev15 (‘-gappyout’) or left untrimmed [44]. Nucleotide alignments were generated with *trimAl* ‘-backtrans’, utilizing aligned amino acid sequences as guides (electronic supplementary material, data files).

Phylogenetic relationships were examined across Isopoda using two different approaches to tree building. Firstly, a supermatrix approach; genes were concatenated in each dataset with *catfasta2phyml.pl* (https://github.com/nylander/catfasta2phyml) then analysed by maximum likelihood (ML) in *IQ-TREE* v. 2.2.0.3 [45. For the amino acid datasets, an initial model selection was performed between four amino acid substitution matrices (‘-m MFP -mset JTT,WAG,LG,Q.insect’), alongside all across site variation and base frequency models available in *IQ-TREE* [46,47]. A preliminary analysis under the best-fit model to create a guide-tree, preceded a final thorough analysis with 1000 ultrafast bootstraps, selected rate variation parameters and a 20 component (C20) profile mixture model [48,49]. Posterior mean site frequency (PMSF) models in *IQ-TREE* are rapid ML approximations to the CAT model of Le *et al*. [50 ], allowing for site-specific variation in amino acid preference across protein-coding sequences (see electronic supplementary material, table S2 for details). For nucleotide-coding alignments, three partitions were analysed: all nucleotides, first and second codon positions, and third codon positions only (to examine the effects of substitutional bias in synonymous sites). Model selection followed by ML analysis under the best-fit model was performed for each (electronic supplementary material, table S2).

A second phylogenetic approach is to generate a summary species tree from individual gene trees, to incorporate any underlying discordance between individual genes. A summary tree was calculated with *ASTRAL* v. 2 [51]. Gene trees, and best model parameters, were estimated in *IQ-TREE2* [52]. Congruence between the supermatrix tree and individual gene tree topologies was assessed with concordance factors in *IQ-TREE* [53]. Gene (GCF) and site (SCF) concordance factors (defined as the percentage of genes or sites that agree with each node) were calculated from individual gene trees and sites in the alignment. Finally, to establish whether the main topology was significantly preferred to any alternate topologies, ML hypothesis testing with approximately unbiased and Shimodaira–Hasegawa (SH) tests were performed in *IQ-TREE* [54]. Alternative topologies included the *ASTRAL* summary tree, alternate placements of *Limnoria*, and topologies examining whether Diplocheta and Tylida were closer to marine isopods (electronic supplementary material, figure S3).

### (b) Marker-gene dataset

A second dataset of traditional phylogenetic ‘marker genes’ was assembled to investigate relationships observed in earlier isopod molecular studies. In total, 11 genes were collated across 148 isopods and 12 outgroups from Genbank (https://www.ncbi.nlm.nih.gov/). These comprised 18S and 28S nuclear rRNA subunits, nuclear protein-coding genes (Na/K ATPase alpha subunit, NAK; phosphoenolpyruvate carboxykinase, PEPCK; histone subunit 3, H3; and intestinal fatty-acid binding protein 2, IGFBP), 12S and 16S mitochondrial rRNAs, and mitochondrial protein-coding genes (COI; NADH dehydrogenase subunit 4, ND4 and cytochrome b, CYTB). Genbank sequences were supplemented by nuclear protein-coding sequences from the isopod genomes and transcriptomes (electronic supplementary material, table S1). *BLAST* (Basic Local Alignment Search Tool) databases were built for contigs with *BLAST* v. 2.13.0 ‘makeblastdb’, queried with isopod NAK, PEPCK, H3 and IGFBP sequences, and top-hit contigs (e-value < 1 ×10^−50^) selected for alignment [55]. Coding sequences were extracted from longer genomic contigs with *MetaEuk* v. 6.a5d39d9, using translated amino acid sequences as reference [56]. To maximize coverage across the marker-gene dataset, in some cases data from two species in the same genus (and in a few cases, the same family) were combined and treated as a single taxon. While such composite taxa are not optimal, there is precedence in supermatrix analyses, and simulations have shown it is better for phylogenetic estimation than a greater proportion of missing data ([57], but see references therein). Care was taken to avoid generating composite taxa where genera might be non-monophyletic (electronic supplementary material, table S3).

As base composition heterogeneity across taxa can add systematic error to analyses, particularly in third codon positions of protein-coding genes [58], all codon positions were assessed for base heterogeneity (‘statefreq’) in PAUP*4.0b10, which compares observed versus expected base composition with a χ^2^ test [59]. Any partitions with significant heterogeneity were re-coded; purines (G, guanine and A, adenine) to R and pyrimidines (C, cytosine and T, thymine) to Y [58]. Data were then retested; RY-recoded codon partitions still suffering from significant composition bias were excluded (electronic supplementary material, table S4). χ^2^ base composition tests were also calculated in *IQ-TREE*.

Sequences were aligned in *MAFFT*, with any misalignments manually adjusted. Non-alignable hypervariable regions in rRNA genes were excluded (all alignments including excluded unaligned regions are provided in electronic supplementary material, data files). A fully automated alignment and trimming approach was also compared; for these, raw *MAFFT* alignments were trimmed with *trimAl*. For each approach, the 11 genes were concatenated into a supermatrix, and ML phylogenetic analyses performed in *IQ-TREE*. The best substitution model for each partition of 18 codon positions and four rRNA sequences was determined (electronic supplementary material, table S4), followed by ML analysis under edge-linked best-fit models for each partition with 1000 ultrafast bootstrap replicates. For this analysis, Isopoda was constrained as monophyletic, due to the unexpected placement of several outgroups and one ingroup taxa. A separate ML analysis was also performed on the 18S alignment (under the best-fit model ‘SYM+R6’) to compare its topology against that of the whole dataset. Finally, to examine nucleotide composition and heterotachy (variable substitution rates between species) across sites in 18S, two further analyses were performed: 18S with RY-recoding and 18S under the general heterogeneous evolution on a single topology (GHOST) model of sequence evolution in *IQ-TREE* [60,61].

### (c) Molecular dating

To obtain divergence estimates across Isopoda, molecular dating was performed on the marker-gene dataset with taxonomic constraints from the phylogenomic dataset, in *BEAST* v. 1.10 [62]. Prior distributions on the root and 15 other nodes were applied based on an interpretation of the isopod fossil record; full details of all fossil constraints and divergence times are given in electronic supplementary material, table S5. Separate analyses with log-normal and uniform prior distributions were performed. To best estimate molecular dates for the five suborders without genomic data (i.e. those most subject to bias from 18S), phylogenetic inference in *BEAST* was also informed by two additional topological constraints from the literature. First, constraining ‘Scutocoxifera’ as monophyletic, supported by both morphology and mitogenomes [30,31,33], informed the placement of Phreatoicidea and Gnathiidea. A second assumed monophyly was Sphaeromatidea (Sphaeromatoidea and Seroloidea), supported by morphological characters [1,30]. To reduce the effects of nucleotide bias for Tainisopidea and Anthuroidea, which grouped together potentially erroneously in the ML tree, preferred placements for each clade were first estimated in separate unconstrained *BEAST* analyses (electronic supplementary material, figure S4). Estimated models of sequence evolution were taken from the ML analysis (electronic supplementary material, table S4). Analyses were run under a relaxed molecular clock, with the birth-death model of speciation, for 100 million generations (sampled every 10 000). Tree and clock parameters were linked across partitions (to avoid problems with overparameterization), and a separate mutation parameter, *Nu*, estimated for each. The ucld.mean prior approximated a gamma distribution, with shape and scale parameters of 0.001 and 1000, respectively. All other priors were default *BEAUTI* v.1.10 values. Convergence and effective sampling were assessed in *Tracer* v. 1.7*;* with effective sample sizes >>100. A maximum clade credibility tree was constructed with *TreeAnnotator* v. 1.10 from 32 000 trees sampled in the posterior distribution of four converging runs.

## 3. Results and discussion

### (a) Phylogenomic dataset

**Figure 1.**
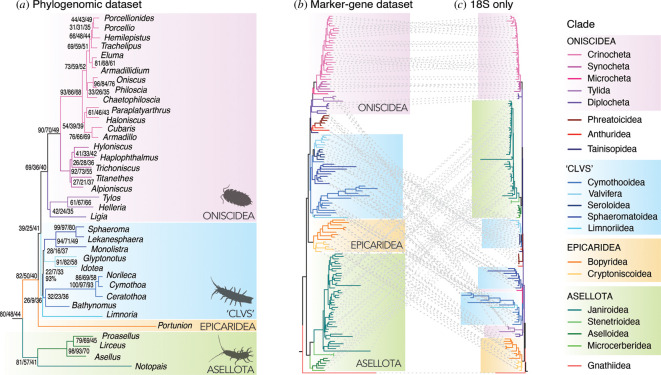
Phylogenies inferred by ML analysis of (*a*) the phylogenomic supermatrix of 970 single-copy orthologues and 36 species, aligned with *FSA* and trimmed with *trimAl,* (*b*) the marker-gene supermatrix of 11 genes across 160 species and (*c*) 18S only from the marker-gene dataset. Nodal values in (*a*) indicate (R to L) concordance factors for sites (SCF), genes (GCF) and GCF for the longest 10% of genes (see main text). Full phylogenies are given in electronic supplementary material, figures S5, 8 and 9. Isopod silhouettes illustrating Asellota: *Proasellus*, I. Frigo; ‘CLVS’: *Pentidotea resicata,* C. Gross; Oniscidea: *Oniscus asellus,* G. Dera are available from https://www.phylopic.org.

Transcriptomes and genomes were assembled for 36 isopods and four pericarid outgroups (electronic supplementary material, table S1). These assemblies were surveyed for single-copy conserved loci (BUSCO *arthropoda_odb10* [41]) present in at least 50% of taxa. The supermatrix alignment of amino acid sequences from these 970 nuclear orthologues produced a resolved, well-supported phylogeny (figure 1*a*, electronic supplementary material, figure S5). Neither the alignment method nor trimming had any effects on topology, which was identical across all datasets with 100% bootstrap support for all nodes except one (see below). Several suborders are recovered as monophyletic, including Asellota and Valvifera, both well supported by morphology [63–65]. The terrestrial isopods, Oniscidea (here comprising Tylida, Diplocheta, Synocheta and Crinocheta), are also monophyletic, contrasting with previous analyses based on nuclear rRNA ([25,26]; electronic supplementary material, figure S2). Tylida and Diplocheta form a monophyletic sister group to Crinocheta and Synocheta. This relationship also differs from previous taxonomic studies of Oniscidea. The widely accepted phylogeny of Erhard [66] recovered Diplocheta as a sister group to the remaining four terrestrial clades, whereas Tabacaru & Danielopol [67] proposed Tylida as a sister group to the rest of Oniscidea, and Schmalfuss [68] recovered a sister relationship between Tylida and Crinocheta. Unfortunately, neither genomic nor transcriptomic data are yet available for *Mesoniscus* (Microcheta) or *Ligidium* (Ligidiidae, Diplocheta), necessary for fully resolving evolutionary relationships across the suborder. Whether this Tylida+Diplocheta sister relationship holds with the addition of more taxa (especially *Ligidium*) or more data (both members of Tylida have below 50% BUSCO completeness) is uncertain (discussed further below).

Relationships within Crinocheta also differ from previous taxonomic studies [21]. There are two well-supported clades within Crinocheta; one consisting of predominantly Northern Hemisphere families (Porcellionidae, Trachelipodidae, Agnaridae, Armadillididae and Oniscidae) together with the palaearctic philoscids, and the second comprising predominantly Southern Hemisphere families (Paraplatyarthridae and Armadillidae) and the Antipodean philoscids. Taxonomists have long considered the family Philosciidae non-monophyletic, but this biogeographical pattern has not previously been reported [21]. This well-supported bifurcation is also observed in the marker-gene dataset, discussed below (figure 1*b*).

Conversely, the suborder Cymothoida, proposed by Brandt & Poore [1] (comprising parasitic Cymothooidea, Epicaridea, Gnathiidea and predatory Anthuroidea) is not monophyletic; the two superfamilies present in this dataset, Cymothooidea and Epicaridea, are recovered separately in the tree (figure 1*a*). This suggests at least two separate origins of parasitism in Isopoda, contrary to previous morphological and nuclear studies, which recover the parasitic clades together [1,25,30,69], confirming recent mitochondrial results [32,33]. Cymothooidea is monophyletic and recovered in a clade with Limnoriidea, Valvifera and Sphaeromatoidea (hereafter referred to as ‘CLVS’), which together form the sister group to Oniscidea. Epicaridea is recovered next to these two clades, followed by Asellota, which branches closest to the root. Interestingly, clade ‘CLVS’ has similarities to the original description of Flabellifera [63] (also referred to as ‘free-living flabelliferans’ in Brandt & Poore [1]) albeit with the inclusion of Valvifera, but confirmation of this requires genomic data for Anthuroidea and Gnathiidea.

The sister relationship between Valvifera and Sphaeromatoidea in this analysis (no genomic data are yet available for Seroloidea) also differs from previous nuclear rRNA phylogenies ([25,29], figure 1*b*), which recover Valvifera with Seroloidea, but Sphaeromatoidea with Cymothooidea, rendering Sphaeromatidea (Sphaeromatoidea and Seroloidea) non-monophyletic. The relationships presented here may thus lend support to the monophyly of Sphaeromatidea, a relatively recent suborder proposed by Wägele [30], and recovered by the analysis of Brandt & Poore [1] though not Brusca & Wilson [31]. An association between Limnoriidea and these marine suborders has been observed in previous taxonomic studies, where Limnoriidea has been recovered with both Cymothooidea and Sphaeromatidea [1,30]. Relationships within ‘CLVS’ in this genomic dataset are similarly uncertain; lower bootstrap support for the sister relationship between Cymothooidea and Valvifera+Sphaeromatoidea may result from the position of Limnoriidea. The topology of the summary species tree estimated from individual gene trees with *ASTRAL* is identical to the supermatrix phylogeny for all datasets except the untrimmed *MAFFT* alignment, where the single difference is that Limnoriidea*,* rather than Cymothooidea, is recovered as a sister group to Valvifera+Sphaeromatoidea (electronic supplementary material, figure S6).

Uncertainty in the placement of Limnoriidea between the gene-tree summary and supermatrix phylogenies is reflected in the concordance factors. GCF and SCF (given at nodes, figure 1*a*) indicate underlying topological variation and are useful statistics for large genomic datasets, where bootstraps quickly reach 100% [53]. Examining concordance factors for each node shows that while some have little discordance (e.g. Crinocheta or Asellota) others have much lower GCF and SCF values (e.g. within ‘CLVS’). Discordance between gene trees can arise due to insufficient and/or conflicting phylogenetic signal. Sequentially removing the shortest genes from the dataset (potentially containing the least phylogenetic signal) reduces the proportion of polyphyletic gene trees and increases GCF values for almost all nodes across the tree, including Oniscidea (electronic supplementary material, figure S7). However, for some nodes (e.g. Diplocheta+Tylida or ‘CLVS’) this trend is less distinct, and GCFs remain low despite the removal of shorter genes, potentially indicating lower and/or conflicting signal at nodes. Tests of topology show a similar result (electronic supplementary material, table S6); across all tests, a sister relationship between Limnoriidea and Valvifera+Sphaeromatoidea could not be significantly rejected compared with the ML tree. Two additional topologies were not rejected by the SH test: one where Diplocheta is the sister group to the remaining Oniscidea (e.g. Erhard [66]), and the second with this clade and a sister relationship between Limnoriidea and Valvifera+Sphaeromatoidea (electronic supplementary material, figure S3*a*, S3*i*, table S6). Importantly, all hypotheses proposing non-monophyly of Oniscidea were significantly rejected across all tests.

While the lower resolution for these nodes might simply be improved with increased taxonomic and genomic sampling (*Limnoria* has below 50% BUSCO completeness), discordance or uncertainty at nodes may also be caused by biological phenomena such as ancient rapid radiation. The divergence of several suborders in quick succession over a short evolutionary timeframe can lead to conflicting signal, through incomplete lineage sorting, and obscured phylogenetic signal, if short internal branches containing few informative substitutions are overwritten by subsequent substitutions in long tips, making phylogenetic resolution more difficult [70]. Long tips and short internal branches are evident at the base of ‘CLVS’ (and potentially Oniscidea, figure 1*a*). Resolving such nodes is exacerbated by missing data and taxa, and additional factors, e.g. excessive substitution rate variation between species (potentially problematic for *Limnoria* and early branching oniscids, see below).

### (b) Marker-gene dataset

The ML analysis of a dataset with much denser taxonomic sampling across 11 phylogenetic marker genes was also performed. This marker-gene dataset contains 148 isopods, across 9 of 11 suborders, with 12 outgroup taxa (electronic supplementary material, table S2). The phylogeny from this dataset is remarkably similar to that of the phylogenomic dataset (figure 1*a, b*; electronic supplementary material, figures S5, S8, S9 and the recent mitogenome phylogeny of Zou *et al*. [33]; electronic supplementary material, figure S2). In both phylogenies in this study, Asellota is recovered nearest the root, followed by Epicaridea, and both contain a clade comprising monophyletic Oniscidea and clade ‘CLVS’. However, there are notable differences within ‘CLVS’. In the marker-gene dataset, *Bathynomus* (Cirolanidae) is not recovered with Cymothooidea but is instead a sister group to Seroloidea and Valvifera (100% bootstrap support), rendering both Cymothooidea and Sphaeromatidea non-monophyletic (figure 1*b*).

To examine these relationships further, a separate ML analysis was performed on 18S, the longest gene in the marker-gene dataset, present in all taxa (figure 1*c*). Notably, areas that differ between the marker gene and phylogenomic topologies appear to directly reflect the 18S phylogeny, which may suffer from long-branch attraction artefacts. In the 18S phylogeny, clade ‘CLVS’ is split; *Bathynomus*, Seroloidea and Valvifera form a strongly supported monophyletic clade nested within Isopoda, whereas Cymothooidea, Sphaeromatoidea and Limnoriidea are recovered nearer the root, close to parasitic Epicaridea and Gnathiidea, littoral oniscids Ligiidae (Diplocheta) and Tylida, and *Mesoniscus* (Microcheta). This renders not only Cymothooidea and Sphaeromatidea non-monophyletic but also Oniscidea; the remaining oniscids, Crinocheta, Synocheta and Ligiididae (Diplocheta) form a monophyletic sister group to Asellota.

Robust testing for long-branch attraction, particularly in rRNA sequences, is challenging [71]. However, consistent with long-branch artefacts, is the observation that both marine and terrestrial isopods recovered near the root have much longer branch lengths than their closest relatives elsewhere in the tree (figure 1; electronic supplementary material, figures S8, 9). In addition, many of the long-branched marine taxa near the root are parasitic, and parasites may have faster substitution rates [72], whereas the shorter branched marine taxa nested in the tree are from deep-sea or cold-water habitats, potentially resulting in slower rates [73]. Finally, many relationships in the 18S phylogeny contradict not only the phylogenomic phylogeny but also well-accepted taxonomy. The placement of several suborders lacking nuclear protein-coding sequences (and genomic data) that are similar between the 18S and marker-gene phylogenies could therefore be incorrect, e.g. a well-supported clade containing Anthuroidea, Tainisopidea and Phreatoicidea nested within the tree, and the placement of Gnathiidea at the root, or outside Isopoda if the topology is unconstrained [1,7,30,31].

Incorrect phylogenetic estimation can arise due to molecular bias (e.g. nucleotide composition or GC-skew in isopod mitogenomes [32,33]). Base composition tests indicate that while several members of Cymothooidea and one asellotan failed individual composition test (electronic supplementary material, table S3), there is no significant composition bias across the whole of 18S (unlike third codon positions; electronic supplementary material, table S4). Furthermore, re-analyses with RY-recoding or under the GHOST model in *IQ-TREE* did not produce meaningful changes to the topology (electronic supplementary material, figures S10, 11). However, ML analysis on third codon positions from the phylogenomic dataset (which can suffer from nucleotide biases such as saturation and base composition) produced a topology with similarities to the 18S tree (electronic supplementary material, figure S12).

These findings suggest that nuclear rRNA genes cannot provide sufficient phylogenetic signal to evaluate higher level isopod relationships. Furthermore, long-branch attraction in 18S may be responsible for generating previous well supported but ultimately incorrect higher level phylogenetic groupings, including the recovery of non-monophyletic Oniscidea and a single origin of parasitism in isopods [24–29,69]. While phylogenetics traditionally relied on nuclear rRNAs prior to the widespread availability of genomic data, studies have demonstrated 18S can be unreliable in arthropods due to nucleotide composition and/or aberrant substitution rates [74,75]. Underlying bias in isopod 18S sequences does not seem to result from differences in base composition (e.g. isopod mitogenomes), but rather from differences in the substitution rate. Lifestyle and life-history traits have been shown to affect species’ substitution rates [27,72,73,76]. Multiple transitions between different environments and lifestyles over isopod evolutionary history could make differences in rates more pronounced.

### (c) Molecular dating and biogeography of Isopoda

**Figure 2.**
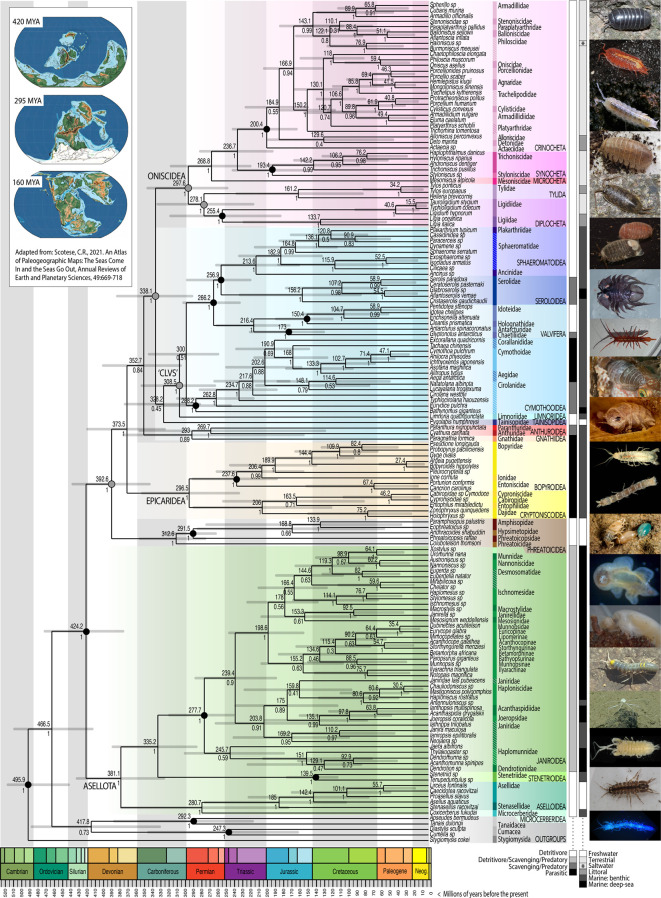
Dated phylogeny from *BEAST* analysis of 11 marker genes with constraints from the phylogenomic dataset (grey circles at nodes) and 16 fossils (black circles). Median divergence estimates in millions of years (values above nodes) and posterior probability (below). Current families are shown with vertical-coloured bars, diagonal hatching indicates non-monophyly in this analysis. Predominant feeding behaviour and habitat are shown in grey scale bars. Inset (left) depicts three world maps at different time periods: late Silurian, 420 Mya; early Permian, 295 Mya; late Jurassic, 160 Mya; adapted from [77]. Images illustrate (*a*) Crinocheta, *Armadillidium vulgare* © W. Maguire; (*b*) Synocheta, *Androniscus dentiger* © W. Maguire; (*c*) Microcheta, *Mesoniscus graniger* © L. Kováč & P. Luptacik; (*d*) Tylida, *Helleria brevicornis* © T. Hughes; (*e*) Diplocheta, *Ligia oceanica* © W. Maguire; (*f*) Sphaeromatoidea, *Sphaeroma serratum* © W. Maguire; (*g*) Seroloidea, *Brucerolis* sp. © A. Hosie; (*h*) Valvifera, *Stenosoma lancifer* © J. Thomas Thorpe; (*i*) Cymothooidea, *Anilocra* sp. © S. Trewhella; (*j*) Limnoriidea, *Limnoria quadripuncata* © S. Trewhella; (*k*) Tainisopidea, *Tainisopus* sp. © G.D.F. Wilson; (*l*) Anthuroidea, *Cyathura carinata* © S. Trewhella; (*m*) Gnathiidea, *Gnathia maxillaris* © S. Trewhella; (*n*) Epicaridea, Cryptoniscoidea *Hemioniscus balani* © P. Adkins; (*o*) Epicaridea, Bopyroidea**,** *Athelges paguri* © J. Thomas Thorpe; (*p*) Phreatoicidea, *Phreatoicus sp.* © G.D.F. Wilson; (*q*) Janiroidea, *Munnopsidae* sp*.* © National Oceanic & Atmospheric Administration (NOAA); (*r*) Stenetrioidea, *Stenetrium* sp*.* © G.D.F. Wilson; (*s*) Aselloidea, *Asellus aquaticus* © W. Maguire; (*t*) Microcerberidea, *Texicerberus* sp*.* © B. Schwartz. (Further information is given in electronic supplementary material, figure S1.)

To examine the timing of evolutionary transitions across Isopoda, a dated phylogeny was estimated from the marker-gene dataset in *BEAST* with 16 fossil calibrations (figure 2, electronic supplementary material, table S5). To limit the effects of 18S nucleotide bias, while maximizing the number of isopods in this analysis, topological constraints were applied from the phylogenomic dataset and taxonomic literature. Consequently, estimated relationships differ from the marker-gene phylogeny (figure 1*b*, electronic supplementary material, figure S8). Molecular dates were slightly older when prior distributions for fossil constraints approximated uniform, rather than log-normal distributions (electronic supplementary material, figure S13). Placing isopod evolution in a temporal and biogeographic framework generates several interesting hypotheses regarding species distributions, vicariance and the timing of evolutionary transitions.

The earliest divergence, between mysid and mancoid peracarids, dates to between the late Ordovician and mid-Cambrian, approximately 496 (446–521) million years ago (Mya). The earliest divergence within Isopoda, between suborders Asellota and Phreatoicidea, dates to approximately 424 (378–475) Mya, between the Devonian and Ordovician. Divergence estimates across the tree suggest that while the earliest representatives of each suborder were likely around before the Permian mass extinction of approximately 250 Mya, most modern families diversified after this, as the Tethys Sea was newly forming (approximately 250 Mya [78]) and Pangaea began to separate (approximately 200 Mya [79]).

#### (i) The earliest Isopoda: freshwater relicts and deep-sea radiations

Despite being one of the earliest diverging isopod suborders, the asellote fossil record is sparse. The first fossil constrains the appearance of Asellota to at least the late Triassic (electronic supplementary material, table S5), though it is likely much older. Here, median divergence dates are younger than previously published estimates [25,28,80]. The earliest divergence, separating Aselloidea and Microcerberidea from Stenetrioidea and Janiroidea, dates to between the Silurian and Carboniferous, approximately 335 (328–435) Mya. The subsequent divergence between Microcerberidea and Aselloidea dates to approximately 281 (174–380) Mya. Microcerberidea are tiny interstitial marine and freshwater isopods with a Tethys-relict distribution. Marine species were likely widespread along the shores of the Tethys, before its regression during the Mesozoic, as demonstrated by the distribution of freshwater taxa in groundwater sources once part of an ancient Tethyan shoreline [81,82]. The divergence between Aselloidea and Microcerberidea should therefore be considerably older than the break-up of Pangaea (240–200 Mya [79]) and therefore biogeographic patterns align better with upper estimates in the late Devonian or early Carboniferous. Transitions between marine and freshwater habitats likely occurred many times in isopod evolution, through similar marine transgressions and regressions (early on, creating whole freshwater suborders, e.g. Phreatoicidea, Tainisopidea, Calabozoidea as well as more recently, within families, e.g. Sphaeromatidae and Cirolanidae [83]). In Asellota, transgressions may have occurred separately on both hemispheres; members of Aselloidea are almost all freshwater and found across the Northern Hemisphere, whereas freshwater Gnathostenetroidoidea have a Gondwanan distribution [82]. The first appearance of Aselloidea may also be closer to upper divergence estimates, approximately 185 (103–277) Mya. However, within Asellidae, the divergence between North American (*Caecidotea* and *Lirceus*) and Eurasian species (*Asellus* and *Proasellus)*, approximately 101 (47–169) Mya, pre-dates the North Atlantic Split.

Relationships within Janiroidea are largely consistent with previous studies, supporting four deep-sea transitions [28,84]. However, the timing of these radiations is debated; while it has been argued that deep-sea Asellota are evolutionarily ancient [85], past oceanic anoxic events may have made the deep sea uninhabitable during the Mesozoic. The molecular dates estimated here suggest at least two transitions from shallow water (Acanthaspidiidae, approximately 98 (17–123) Mya and Haploniscidae, approximately 81 (37–135) Mya) may have occurred after the most recent (‘Bonarelli’ approximately 93 Mya and ‘Selli’ approximately 120 Mya) anoxic events during the Cretaceous. Conversely, transitions to deep-sea life occurring in Munnopsidae (the largest radiation containing the majority of deep-sea families) approximately 199 (155–247) Mya, and Dendrotionidae+Haplomunnidae approximately 151 (84–224) Mya, likely occurred before Cretaceous and Jurassic (Toarchian approximately 183 Mya) anoxic events, but after the Permian mass extinction anoxic event (approximately 250 Mya).

Unfortunately, this dataset cannot resolve whether Asellota or Phreatoicidea are the earliest branching isopod suborder. The earliest fossil isopods appear to be marine phreatoicids, with a wider distribution than today [86]. Divergence estimates for Phreatoicidea may date the origin of freshwater taxa from a marine transgression event in the Southern Hemisphere approximately 313 (277–357) Mya. Extant phreatoicids have a relict Gondwanan distribution, with species in New Zealand, Australia, India and South Africa likely dating to the break-up of Gondwana. For example, members of Hypsimetopidae can be found in both Australia and India; the divergence within this family should date to at least 130 Mya, when India separated from Gondwana [87]. Divergence estimates between Amphisopidae and Hypsimetopidae, approximately 169 (55–285) Mya, may therefore align with geological dates.

#### (ii) Multiple origins of parasitism from free-living marine Isopoda

Divergence estimates for Scutocoxifera fall between the mid-Carboniferous and Silurian-Devonian boundary, approximately 374 (327–422) Mya. In this analysis, Epicaridea diverges before a clade containing Gnathiidea and Anthuroidea. While morphological characters support a sister relationship between both Gnathiidea and Anthuroidea [1], and Gnathiidea and Epicaridea [31], current taxonomy places Gnathiidea and Anthuroidea with Cymothooidea in Cymothoida. While support for the sister relationship between Gnathiidea and Anthuroidea (0.89 posterior probability, PP), and for their placement outside of Oniscidea+‘CLVS’ (0.84 PP) is not maximal, neither group appear to be closely associated with Cymothooidea. Posterior probabilities are similar for the placement of Tainisopidea inside Oniscidea+‘CLVS’ in the unconstrained analyses (0.83 PP, electronic supplementary material, figure S4), though equivocal for whether Tainisopidea is closer to ‘CLVS’ or Oniscidea. Previous taxonomic analysis has suggested that Tainisopidea might be related to ‘Flabellifera’, whereas the freshwater subterranean suborder Calabozoidea (without sequence data) may be closer to Oniscidea [82]. Clearly, further genomic confirmation is needed, but such findings suggest the higher level taxonomy of Isopoda may require revision; either through reinstating suborder status for Cymothooidea, Gnathiidea and Anthuroidea, alongside Epicaridea, or by creating a new suborder containing Cymothooidea, Limnoriidea, Valvifera, Sphaeromatidea and potentially Tainisopidea.

Fossil swellings, characteristic of isopod parasite infection, can be seen in decapods from the Late Jurassic (electronic supplementary material, table S5). In Epicaridea, the divergence between decapod parasites, Bopyroidea, and parasites of other crustaceans, Cryptoniscoidea, dates to approximately 296 (235–355) Mya. The earliest epicarids may therefore have appeared just before the earliest scavenging cirolanids in Cymothooidea, approximately 288 (245–337) Mya, but well before obligate fish parasites in Cymothoidae, approximately 103 (67–141) Mya. These dates suggest that cymothooids may have evolved alongside their teleost fish hosts (between the early Triassic and late Cretaceous [88]). In comparison, extant Epicaridea may have diverged sometime after the first appearance of decapods and other crustacean hosts in the Palaeozoic oceans [36,89].

The appearance of clade ‘CLVS’, corresponding to the split between Limnoriidea and the remaining suborders (as in the phylogenomic dataset), dates to the Carboniferous, approximately 309 (265–355) Mya. Sphaeromatidea (Seroloidea and Sphaeromatoidea) and its divergence from Valvifera both date to the early Triassic. Sphaeromatoidea and Valvifera date to between the Permian and mid-Jurassic, approximately 216 (172–262) and approximately 214 (161–263) Mya, respectively. Northern and Southern Hemisphere representatives in all sphaeromatid and valviferan families (except the arcturids, split into Northern Hemisphere Arcturidae and Southern Hemisphere Antarcturidae and related families [64]) could suggest both groups were widespread throughout the Tethys during the Triassic, and familial divergences should date to the break-up of Pangaea. These Triassic dates also contrast to younger divergence estimates, approximately 156 (84–233) Mya, for Seroloidea, which has a Gondwanan distribution and likely origin [90,91]. Within Serolidae, the divergence between South American and Antarctic serolids, approximately 59 (16–116) Mya, is slightly older than geological estimates for the opening of Drake’s Passage (approximately 49 Mya [92]), which restricted dispersal between the continents.

#### (iii) A single transition to land in terrestrial Isopoda

Estimating the divergence of Oniscidea dates the origins of isopod terrestriality. The earliest fossil oniscids are from the mid-Cretaceous, approximately 105 Mya (electronic supplementary material, table S5). In this analysis, Oniscidea dates to the Carboniferous–Permian boundary, approximately 298 (249–348) Mya, suggesting isopods transitioned to land considerably later than other terrestrial arthropods. Molecular estimates for hexapods, myriapods and arachnids fall between the Ordovician [35] up to the Silurian or Cambrian [93], alongside the emergence of terrestrial plants.

Early oniscids may have been littoral, similar to *Ligia, Tylos* and the earliest branching families within Crinocheta (Scyphacidae, Actaeciidae, Alloniscidae and Detonidae). Evolutionary forays further inland appear to have taken place multiple times, in each terrestrial group. Outside Crinocheta, terrestrial isopods are highly dependent on moisture, either through proximity to sources of surface water (e.g. riparian *Ligidium* (Ligidiidae, Diplocheta)), or through behavioural and physiological adaptations to life underground (e.g. leaf-litter dwelling *Helleria* (Tylida) burrows below ground during droughts [94]). *Mesoniscus* and many Synocheta are subterranean, displaying adaptive, potentially convergent, traits for this lifestyle [21]. The first transitions inland from littoral habitats may have occurred in the late Permian (potentially on northern Pangaea as the south was frozen; inset figure 2). *Mesoniscus* (Microcheta), found solely in Europe, is estimated to have diverged from Synocheta and Crinocheta approximately 269 (221–319) Mya ago. Within Diplocheta, the divergence between globally distributed Ligiidae and Northern Hemisphere Ligidiidae dates to approximately 255 (184–319) Mya. The divergence in Tylida between *Tylos* and forest-dwelling *Helleria* is more recent, approximately 161 (82–238) Mya.

Divergence estimates in Synocheta between southern Styloniscidae and northern Trichoniscidae, approximately 193 (148–242) Mya, may date to the break-up of Pangaea. Within Crinocheta, the earliest branching littoral families are found across the Southern Hemisphere and tropics. The divergence between the predominantly Northern and Southern Hemisphere clades, approximately 167 (131–203) Mya, is younger than estimates for Synocheta. While this could simply be underestimated, notably no native Northern Hemisphere crinochetans are found in North America [95] (e.g. compared with distributions for Asellidae, Ligidiidae, Trichoniscidae and many insect taxa) or throughout Asia (which has Southern Hemisphere Crinocheta), only more recent human-mediated introductions. This could suggest localized extinction, or that Crinocheta were not widespread across Laurasia before its break-up. Indeed, considering that many Northern Hemisphere crinochetan families are found in Europe and North Africa, these estimates might correspond better to the split of South America and Africa from the rest of Gondwana (beginning approximately 170 Mya [87]). However, the palaeobiogeography of Africa with Laurasia after separation from Gondwana is complex [96]. Increased genomic sampling across Crinocheta, particularly in Africa, may better clarify the origins of Northern Hemisphere taxa, and other unexpected biogeographic patterns, e.g. the widespread Mediterranean distribution of *Armadillo officinalis* (of predominantly Southern Hemisphere Armadillidae). Increased sampling may also elucidate other important aspects of oniscid evolution, e.g. the origin of pleopodal lungs. The phylogeny here suggests a single origin in the Northern Hemisphere, approximately 106 (78–138) Mya, with the five more complex pleopods of Agnaridae and Trachelipodidae potentially evolving from five simple uncovered lungs, similar to *Oniscus* and *Philoscia* [19]. Pleopods appear to have arisen multiple times in the Southern Hemisphere; ancestral to Armadillidae (and potentially Eubelidae, lacking sequence data) approximately 86 (44–138) Mya, and Philosciidae+Balloniscidae, approximately 51 (18–92) Mya [19,20]. All these dates fall towards the latter part of the Cretaceous and early Palaeogene, coinciding with the climate becoming cooler and drier [97].

## 4. Conclusions

Terrestrial isopods have long been considered an example of how marine taxa might transition to life on land. However, early molecular phylogenies suggested multiple origins of terrestriality within Isopoda [25,26]. In contrast, this study finds compelling evidence for a monophyletic Oniscidea, as originally proposed by morphology [21,30,31,67–69], and consistent with a single origin of terrestriality in isopods. This is the first study to use nuclear genomic and transcriptomic data to investigate and date evolutionary relationships across Isopoda, finding agreement between different phylogenetic approaches and data types, and concordance across genes. Analysis of a taxonomically rich marker-gene dataset indicates that previous studies may have been misled by long-branch attraction artefacts in nuclear rRNA sequences. Additionally, the relationships presented here are largely similar to those recovered in previous mitogenome studies and a recent broader crustacean phylogenomic analysis [33,36]. This study shows that while nuclear orthologues are both suitable and sufficient for resolving phylogenetic relationships in Isopoda, greatly increasing taxonomic sampling with genomic data is essential to better understand isopod evolution. Obtaining crucial missing taxa within Oniscidea (e.g. *Mesoniscus* (Microcheta) and *Ligidium* (Diplocheta)), and deeper sampling across the rest of Isopoda (Phreatoicidea, Gnathiidea, Anthuroidea, Seroloidea, and elusive freshwater subterranean Tainisopidea and Calabozoidea) will be important to confirm the findings presented here.

Isopoda have undergone numerous evolutionary transitions, enabling their radiation across almost all environments on Earth. Sequencing across Isopoda will elucidate not only the number and timing of transitions, between marine and freshwater habitats, benthic and deep-sea ecosystems, free-living and parasitic lifestyles, and aquatic and terrestrial environments (and back again), but also which genes are involved. While there may have been just one transition to terrestrial life, pleopodal lungs have likely evolved several times. Genomic analysis will reveal whether similar transitions rely on the same underlying genetic machinery, or if novel pathways are adopted. Through advances in sequencing technologies, and large-scale genomic projects (e.g. [98]), high-quality genomes are increasingly becoming available. Widescale genome production will allow the evolutionary history and genetic basis of adaptation to diverse environments to be explored across Isopoda.

## Data Availability

Supplemental data files and scripts for this manuscript are available in [99]. Supplementary material is available online [100].
